# Beating tumour drug resistance: “Lamarckian” induction in the spotlight

**DOI:** 10.1111/pcmr.12744

**Published:** 2018-11-06

**Authors:** Carla Daniela Robles‐Espinoza

In other words, both a “Darwinian” selection driven by somatic mutations and a “Lamarckian” induction driven by epigenetic changes and transcriptional plasticity can contribute to the establishment of drug resistance

Drug resistance is a major problem in cancer treatment. In cutaneous melanoma, a large fraction of tumours carry *BRAF* ^V600E^ or *BRAF* ^V600K^ mutations (hereinafter referred to as *BRAF* ^V600E/K^), which result in activation of the mitogen‐activated protein kinase (MAPK) pathway, sustaining cancer cell proliferation and survival. In 2011, a landmark phase III study demonstrated that vemurafenib, a drug specifically targeting cells with *BRAF* ^V600E^ mutations, significantly improved progression‐free survival over the conventional chemotherapy drug dacarbazine (Chapman et al., 2011). However, resistance soon evolved in patients treated with this drug and with another *BRAF* ^V600E^‐targeting drug, dabrafenib, due to continued activation of the MAPK pathway through downstream targets of BRAF such as MEK. In 2015, another multicentre, double‐blind clinical trial demonstrated the improved efficacy of combined therapy with dabrafenib and trametinib, resulting in concomitant BRAF and MEK inhibition, over treatment with dabrafenib alone. However, about half of patients treated with this combination drug therapy (BRAFi/MEKi) still progress after 12 months (Long et al., 2016; Welsh, Rizos, Scolyer, & Long, 2016). Why does this resistance develop, and where does it come from?

One of our current paradigms posits a mutational model for the acquisition of tumour drug resistance, in which cells either originally had, or acquire upon treatment, additional genetic alterations that provide them with the ability to outgrow their neighbours, become insensitive to therapy or escape immune surveillance. Accordingly, studies of the acquisition of resistance in BRAFi/MEKi‐treated tumours have shown reactivation of the MAPK pathway through mechanisms such as *BRAF* amplification, mutations of *MEK* and oncogenic mutations in *NRAS* (Long et al., 2014). However, it seems that resistance keeps emerging even as we sequentially target somatic driver mutations. In this context, exciting new research has suggested that this mutational model might not represent the whole story—indicating that to achieve durable responses in cancer treatment, we might need to start looking elsewhere.

In their remarkable study, Rambow *et al* set out to study the mechanisms of acquisition of therapy resistance in *BRAF* ^V600E/K^‐mutant melanomas treated with BRAFi/MEKi. They focused on extensively characterizing minimal residual disease (MRD), defined as the subpopulation(s) of cells within a tumour that confer resistance upon treatment and eventually drive relapse. In order to do this, they established patient‐derived xenograft (PDX) models of *BRAF* ^V600E/K^‐mutant melanomas and exposed mice to BRAFi/MEKi treatment with the dabrafenib/trametinib combination. They observed three phases of treatment response: A rapid tumour shrinkage phase (phase 1), a phase where the tumour became impalpable (phase 2) and finally a phase where the tumour relapsed (phase 3). However, they realized that cells sampled from phase 2, where MRD remained, did not have significantly different genomic profiles from cells taken before tumours were exposed to BRAFi/MEKi. This result suggested, perhaps surprisingly, that MRD was established in this model through non‐mutational mechanisms.

Next, the authors extensively characterized the MRD cell population by single‐cell RNA sequencing and pseudo‐time analysis. They found that drug exposure induced a transcriptional state characterized by intermediate MITF activity and a gene expression profile reminiscent of nutrient‐deprived cells, which they termed “starved‐like melanoma cells,” or SMCs. They observed that these cells could move along a differentiation trajectory to become either a “pigmented” subpopulation, characterized by elevated MITF activity and markers of differentiation and pigmentation, or adopt a de‐differentiated state that can then become either an “invasive” phenotype characterized by the expression of epithelial‐to‐mesenchymal transition markers, or a “neural crest stem cell (NCSC) like” population characterized by high expression of NCSC markers. All these subpopulations are drug‐tolerant, and the authors’ analyses suggest that these states can all co‐exist in MRD. They show regional heterogeneity of these drug‐tolerant cells within tumours as well as great interpatient variability.

The NCSC subpopulation was noteworthy—it increased dramatically even as the tumour was shrinking during drug exposure. Intriguingly, cells seemed to be able to reversibly transition into this state through phenotype switching, as some of the cell lines analysed by the authors did not initially display NCSC markers but did so upon drug exposure, and lost marker expression upon drug removal. This plasticity lent further support to the non‐mutational nature of this phenomenon. Moreover, the increase in the proportion of NCSCs seemed to be a BRAFi/MEKi‐specific effect, as it was not observed in cell lines exposed to other cell proliferation inhibitors. It also seemed to be independent of genetic background and mutational state, as it was seen both in *NRAS*‐mutant and triple wild‐type melanoma cell cultures. The authors also elegantly identified the retinoid X receptor Y (RXRG) as a key driver of the NCSC state through a gene regulatory network analysis and showed that targeting it together with BRAFi/MEKi can significantly delay the development of resistance.

**Figure 1:**
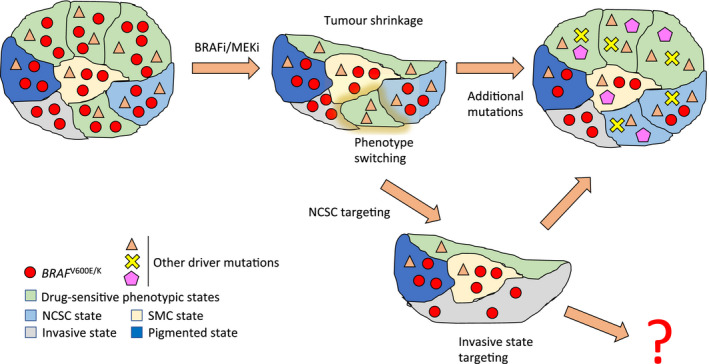
Both mutational and non‐mutational mechanisms for the acquisition of drug resistance can be operational in a tumour. A tumour with *BRAF* ^V600E/K^ mutations is treated with BRAFi/MEKi. The BRAF‐mutated cells in drug‐sensitive phenotypic states die, but cells in the four drug‐tolerant phenotypic states survive treatment. Cells can also undergo phenotype switching to the NCSC state that ultimately gives rise to relapse. Then, additional somatic mutations can be acquired that allow the tumour to grow and resist treatment. If the NCSC subpopulation is targeted, the tumour takes significantly longer to relapse, but eventually does so upon expansion of cells in the “invasive” state. The invasive state could also potentially be targeted. Note that this diagram is over simplistic and that different subpopulations can co‐exist in the same geographical space. NCSC: Neural crest stem cell‐like, SMC: starved‐like melanoma cells

So how do we reconcile these results with our current understanding of the acquisition of drug resistance? A model (Salgia & Kulkarni, 2018) has recently been proposed in which non‐mutational events can lead to an initial drug‐tolerant state, as was observed by Rambow *et al*, followed by the acquisition of somatic mutations that provide cells in this drug‐tolerant state with a growth advantage. In other words, both a “Darwinian” selection driven by somatic mutations and a “Lamarckian” induction driven by epigenetic changes and transcriptional plasticity can contribute to the establishment of drug resistance. The latter phenomenon suggests that a better, “adapted” cell state can be induced by environmental cues and inherited to daughter cells. This model combining mutational and non‐mutational resistance mechanisms would fit well with observations made by Rambow *et al* in their study, which revealed that the NCSC population emerged independently of genetic background and that some phase 3 tumours, after relapse, carried known resistance‐associated mutational events such as de novo mutations in *MEK* and *NRAS* as well as *BRAF* amplifications and splice variants (Figure 1).

Although drug resistance remains a major problem in cancer treatment, we might be getting closer to understanding it. The study by Rambow *et al* provides us with important clues for targeting both the mutational and non‐mutational mechanisms of drug resistance in melanoma—perhaps by BRAFi/MEKi in combination with an RXRG antagonist (RXRi). Obstacles still remain—BRAF/MEK/RXRi‐treated PDX tumours still relapsed (albeit significantly later), and the “invasive” cell population expanded and took over the tumour upon reduction in the NCSC population. Therefore, we might require concomitant targeting of the “invasive” and NCSC tumour subpopulations in addition to MAPK pathway inhibition, or we might need to block the ability of cells within a tumour to undergo phenotype switching in response to drug exposure. Given that this mechanism may represent a generalized path to the acquisition of tumour drug resistance, this significant work brings to light the importance of considering the duality of mutational and non‐mutational mechanisms when devising cancer treatment strategies.
